# Red‐shifted optogenetics comes to the spotlight

**DOI:** 10.1002/ctm2.807

**Published:** 2022-04-07

**Authors:** Tianlu Wang, Siyao Liu, Yun Huang, Yubin Zhou

**Affiliations:** ^1^ Center for Translational Cancer Research Institute of Biosciences and Technology Texas A&M University Houston Texas USA; ^2^ Center for Epigenetics and Disease Prevention Institute of Biosciences and Technology Texas A&M University Houston Texas USA; ^3^ Department of Translational Medical Sciences College of Medicine Texas A&M University Houston Texas USA

Optogenetics has emerged as a powerful technique to precisely manipulate protein activity and cell signalling in space and time since its first introduction to the neuroscience field in 2005^1^, ^2^. The clinical translation of optogenetics has gained momentum by the recent demonstration of a red‐shifted microbial opsin ChrimsonR to partially restore the vision of a blind patient with a single‐dose adeno‐associated virus (AAV) injection into the eyes.^3^ Photosensory modules responding to red/far‐red light offer an ideal solution for future wireless optogenetic therapies due to their deep tissue penetration capability and relatively low phototoxicity. The applications of these red/far‐red light‐switchable optogenetic devices, in theory, can be expanded into clinical domains beyond ophthalmology to encompass neurology, psychiatry, endocrinology, and oncology.

## 1 WHERE WE STAND WITH RED‐SHIFTED OPTOGENETICS

A multitude of red/far‐red light‐triggered optogenetic tools have been created to overcome the limited tissue penetration issue associated with blue photoreceptors. For instance, microbial opsins have been engineered to perceive photons emitting in the range of 500–650 nm, thereby allowing transcranial activation or inactivation of neurons.^2^ In addition, plant‐derived phytochrome B (PhyB) has been engineered to pair with phytochrome interaction factor (PIF) to constitute one of the first efficient red/far‐red light responsive optical dimerization systems. More recently, with endogenous chromophore biliverdin (BV) IXα tetrapyrrole as the light absorber, bacterial phytochrome photoreceptors (BphP1) has been reported to form near‐infrared (NIR) light‐inducible heterodimerization with their natural binding partner PpsR2 or the truncated variant Q‐PAS1^4^ as well as nanobody‐based binders.^5^ These photoreceptors are found to be efficient in gene transcription regulation when ectopically introduced into mammalian cells and animal models. However, their large sizes, limited ranges of photo‐induced dynamic changes, sub‐optimal reversibility, and/or non‐negligible background activation in the dark hamper their further in vivo applications.

Another bacterial photoreceptor, BphS, is able to convert guanosine triphosphate (GTP) into cyclic diguanylate monophosphate (c‐di‐GMP), and subsequently triggers the dimerization of an engineered hybrid transactivator, BldD‐p65‐VP64. Taking advantage of this photochemical feature, the Ye group at East China Normal University developed smartphone‐controlled optogenetically engineered cells to enable semi‐automatic control of blood glucose levels in diabetic mice, which sets the stage for translating cell‐based therapies for diabetes intervention.^6^ In addition, combined with CRISPR/Cas12a^7^ or CRISPR/dCas9^8^, BphS was further harnessed to manipulate target gene expression at endogenous genomic loci. Nevertheless, the requirement of prolonged light illumination and the immunostimulatory effect of c‐di‐GMP make this type of optogenetic device face multiple translational barriers.

## 2 REDMAP AS A MINIATURE OPTOGENETIC SYSTEM

**FIGURE 1:**
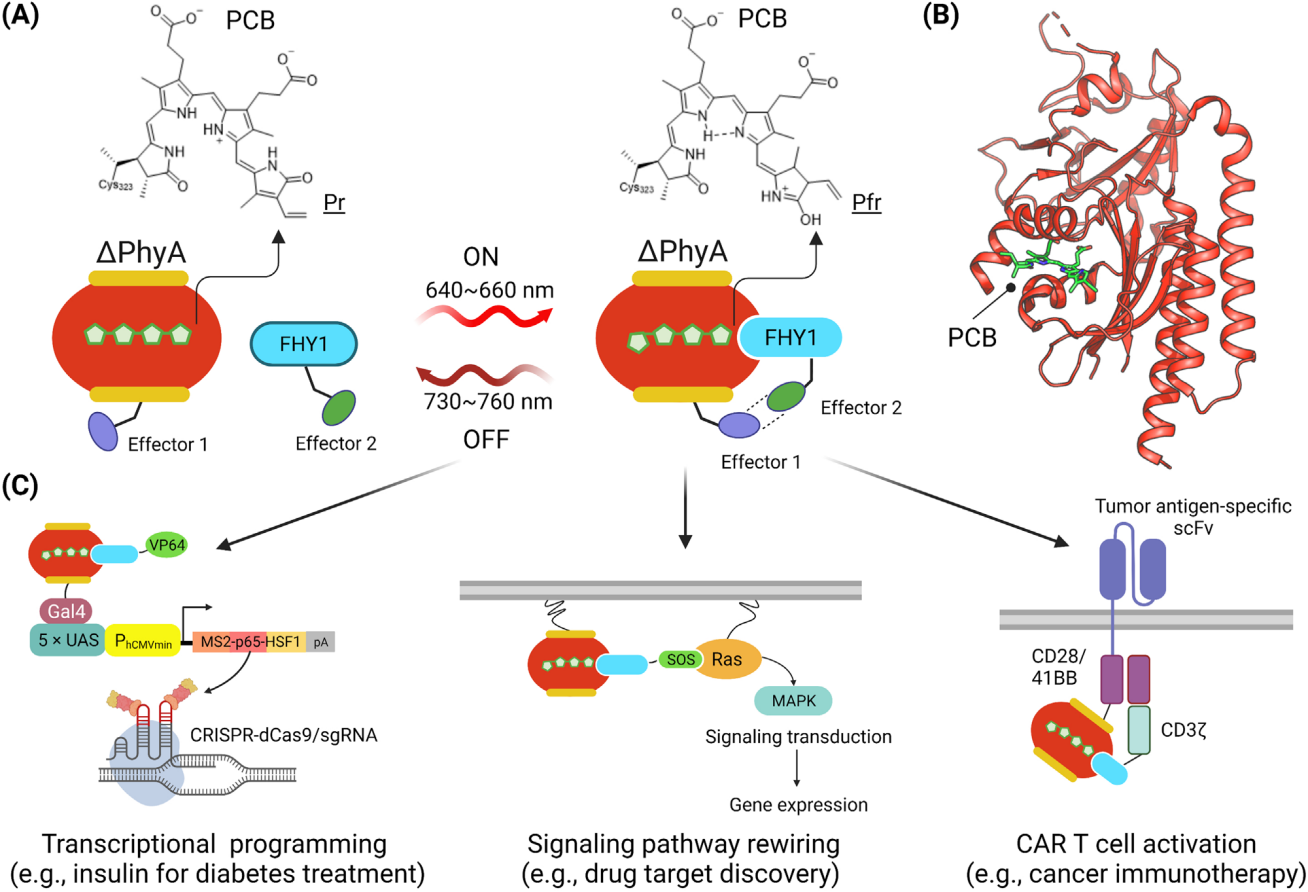
REDMAP for optogenetic applications. The cartoons were created with BioRender.com. (A) The chemical basis and working principle of the two‐component REDMAP system, which contains a minimal N‐terminal light‐sensitive domain from *Arabidopsis* phytochrome A (ΔPhyA) and its binding partner FHY1. Phycocyanobilin (PCB) serves as the light‐absorbing cofactor. Upon red‐light stimulation (640–660 nm), ΔPhyA switches to a Pfr state to interact with FHY1. Under far‐red light illumination (730–760 nm), ΔPhyA reverts to a Pr state and dissociates with FHY1. (B) The three‐dimensional structure of the N‐terminal photosensory module of a plant phytochrome A (red; PDB entry: 6TC7) with its cofactor PCB (green). (C) REDMAP for biomedical applications. (Left) The REDMAP components are fused to effector domains, such as Gal4 and VP64, and further coupled with CRISPR‐dCas9 to drive transgene (e.g., insulin) or endogenous gene expression. (Middle) REDMAP used to photo‐control Son of Sevenless (SOS) translocation toward the plasma membrane to activate MAPK signaling, as well as downstream gene transcription. (Right) The envisioned application of REDMAP to design red light‐activatable chimeric antigen receptor (CAR) T cells for cancer immunotherapy

To tackle the above‐mentioned roadblocks, the Ye group continued to develop a red‐shifted optogenetic devices, termed as REDMAP for red/far‐red light‐mediated and miniaturized PhyA (ΔPhyA)‐based photoswitch^9^ (Figure 1A,B). REDMAP is composed of the photosensory module of *Arabidopsis* photoreceptor PhyA and the shuttle protein far‐red elongated hypocotyl 1 (FHY1). Under red light illumination (*λ*_max_ = 640–660 nm), ΔPhyA adopts a Pfr state and associates with FHY1 within seconds. When exposed to far‐red light (*λ*_max_ = 730–760 nm), ΔPhyA quickly switches to a Pr state to disrupt its interaction with FHY1 (Figure 1A). Compared to the PhyB/PIF combination, the REDMAP system has a compact size of only ∼3.2 kb, and therefore, can be packaged into clinically approved AAV vectors for efficient delivery into rodent livers.^9^ The REDMAP components can be engineered into the yeast Gal4 DNA‐binding domain and the artificial transactivator VP64 (Figure 1C), thereby achieving potent transcriptional activation (>150‐fold) with fast kinetics (∼1 s for activation) and mild light dose (1 mW/cm^2^). In living mammals, REDMAP was shown to enable red light‐inducible expression of insulin in type 1 diabetic mice and rat, as well as transgene expression in rabbits. Most impressively, illumination with red light for only 1 min was sufficient to induce transgene expression in vivo. Likewise, 1 min of far‐red light illumination was able to switch off transgene expression, a desirable feature that will accelerate the future clinical translation. The REDMAP system was further successfully deployed to control the subcellular localization of signaling proteins (e.g., the RasGEF catalytic domain of Son of Sevenless [SOS]) and confer dynamic control over the endogenous MAPK pathway (Figure 1C), as well as a rewired MAPK pathway to control customized transgene expression.^9^ In parallel, REDMAP was coupled with the CRISPR‐dCas9 system to enable transcriptional programming, in which red light‐induced transgene expression of MS2‐p65‐HSF1 could efficiently turn on the expression of user‐defined endogenous genes both in cultured human cells and rodent organs (Figure 1C).

## 3 FUTURE PERSPECTIVES

Optogenetic therapy promises to become a non‐conventional therapeutic modality with minimal invasiveness but high precision for human disease intervention. As a proof of concept, the REDMAP device has shown great success in light‐tunable production of insulin for diabetes treatment in type 1 diabetic mice. Similar applications can be envisioned in the treatment of monogenic disorders, in which the photo‐tunable gene expression and/or the light‐inducible genome editing could curtail or even reverse the disease progression, thereby benefiting millions of patients suffering from sickle cell anemia, cystic fibrosis, Duchenne muscular dystrophy, or Huntington disease. As a low‐hanging fruit, REDMAP‐like devices could be readily coupled with adoptive cell therapy to develop red light‐responsive chimeric antigen receptor (CAR) T cells for cancer treatment (Figure 1C), as recently done by using blue light‐sensitive optical dimerizers when coupled with NIR light‐activatable upconversion nanoparticles.^10^

Moving forward toward translational applications, several caveats associated with REDMAP or similar devices should be kept in mind. First, the plant phytochrome‐based REDMAP relies on phycocyanobilin (PCB) as the light absorber, thereby necessitating the external supply of this exogenous cofactor or the co‐expression of multiple plant genes involved in PCB synthesis. Interestingly, the blue‐green algae spirulina, which can be up‐taken orally as a dietary supplement, might serve as a rich source of PCB, but the effective dose required for phytochrome photoactivation remains be established in human. Second, the immunogenicity and potential genotoxicity of these plant‐derived proteins during long‐term use remain uncharacterized. This risk becomes more prominent if these foreign molecules are introduced at the extracellular side of the plasma membrane. Third, like any other gene therapies, the delivery of genetic materials and tissue targeting specificity still have tremendous improvement space. Recent progress in the development of tissue‐specific AAVs and lipid nanoparticles might offer potential solutions to resolve this issue. Regardless, considering its photo‐tunability, superior reversibility, and unprecedented spatiotemporal resolution, red‐shifted optogenetics might offer a non‐conventional means to develop next‐generation precision medicine, in which the therapeutic window can be personalized according to each patient's own need.

## CONFLICT OF INTEREST

The authors declare no conflict of interest.
